# The increasing likelihood of temperatures above 30 to 40 °C in the United Kingdom

**DOI:** 10.1038/s41467-020-16834-0

**Published:** 2020-06-30

**Authors:** Nikolaos Christidis, Mark McCarthy, Peter A. Stott

**Affiliations:** Met Office Hadley Centre, FitzRoy Road, Exeter, EX1 3PB UK

**Keywords:** Climate change, Attribution, Projection and prediction

## Abstract

As European heatwaves become more severe, summers in the United Kingdom (UK) are also getting warmer. The UK record temperature of 38.7 °C set in Cambridge in July 2019 prompts the question of whether exceeding 40 °C is now within reach. Here, we show how human influence is increasing the likelihood of exceeding 30, 35 and 40 °C locally. We utilise observations to relate local to UK mean extremes and apply the resulting relationships to climate model data in a risk-based attribution methodology. We find that temperatures above 35 °C are becoming increasingly common in the southeast, while by 2100 many areas in the north are likely to exceed 30 °C at least once per decade. Summers which see days above 40 °C somewhere in the UK have a return time of 100-300 years at present, but, without mitigating greenhouse gas emissions, this can decrease to 3.5 years by 2100.

## Abstract (web summary)

The United Kingdom has experienced a strong heat wave in 2019 that set a new temperature record for the country of 38.7 °C. In this study the authors show that under climate change, local temperatures are increasingly likely to exceed 35 °C and 40 °C in the next decades and, hence, summers like the one of 2019 become more frequent.

## Introduction

**Fig. 1:**
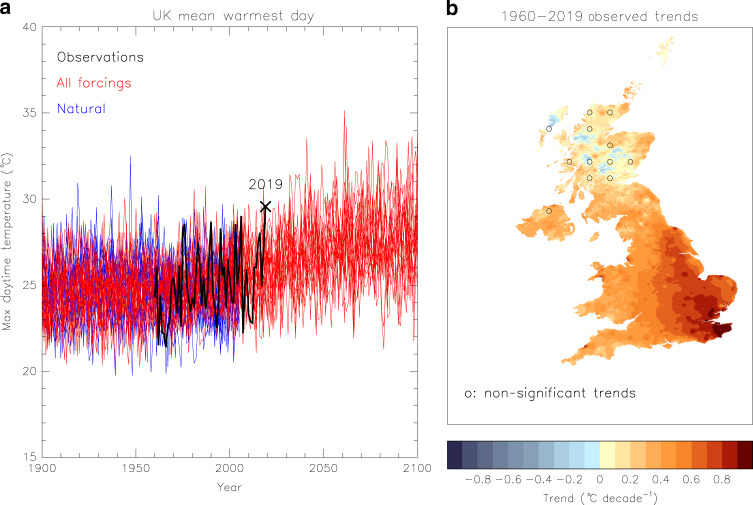
Warmest daytime temperatures (*tx01*) in the UK. **a** Timeseries of the UK mean *tx01* from HadUK-Grid observations (black line), and simulations with 16 CMIP5 models with all climatic forcings (red lines) and natural forings only (blue lines). The observed value in 2019 is marked with a cross. Simulations of future years follow the RCP 4.5 scenario. The model data were bias-corrected to have the same mean during a reference period as the observations. **b** A map of the *tx01* trends during 1960–2019 computed with HadUK-Grid data. Circles mark areas (of ~60 × 60 km) where most grid boxes have trends not significantly different from zero (tested at the 10% level), as determined by a Mann–Kendall test.

Intensification of hot extremes has continued unabated in recent decades^1^, posing a threat to human health^2,3^ and bringing forth a raft of further socio-economic impacts^4,5^. Europe is gearing up for more frequent and intense heatwaves^6^ and while the UK has not yet borne the brunt of extreme continental heat, its summer temperatures are decidedly on the rise^7,8^. Attribution research provides strong evidence that hot extremes are becoming more frequent and intense^9^ under the influence of human-caused climate change^10,11^. The UK summer temperature of 2018 was a joint record, estimated to have become 30 times more likely due to anthropogenic causes^12^. A year later, during a severe heatwave in western Europe^13^, the warmest daily temperature averaged over the UK reached a new peak (Fig. 1a) and the highest temperature in the country ever recorded was registered in Cambridge. These consecutive summer extremes are exposing the UK’s vulnerability to such weather with ensuing impacts highlighted in the media, including a mortality spike in tandem with the 2019 event^14,15^, and a sharp heatwave-driven fall in overseas holiday demand that might have contributed to the collapse of the Thomas Cook travel group^16^. Therefore, the need to understand how the likelihood of extremely hot temperatures is changing under the anthropogenic effect on the climate is pressing and essential to decision-makers planning the UK’s adaptation strategy.

To respond to this need, we compute observed and modelled values of the warmest daily maximum temperature in individual years (*tx01*) and estimate how the likelihood of exceeding extreme thresholds has been changing since 1900 and how it may further change in the remaining of this century under different emission scenarios^17^. We find that the likelihood of extremely warm days in the UK has been increasing and will continue to do so during the course of the century with the most extreme temperatures expected to be observed in the southeast England. The likelihood of exceeding 40 °C anywhere in the UK in a given year has also been rapidly increasing, and, without curbing of greenhouse gas emissions, such extremes could be taking place every few years in the climate of 2100.

## Results

### Observed changes in UK *tx01* extremes

Limitations arising from the spatial resolution of climate models and the coverage of observation stations often prevent attribution studies on local scales and have kept the focus on extremes over larger, sub-continental areas^18^. Although downscaling of model output has occasionally been employed to investigate local events^19^, the lack of reliable observations makes it difficult to evaluate the models. Here, we take advantage of the recent upgrade of the HadUK-Grid dataset^20^ for daily maximum temperature, which now provides observational data on a high-resolution grid of 1 × 1 km. The resolution of the dataset enables us to model the relationship between local and UK mean *tx01*. This simple downscaling technique allows us to estimate changes in the likelihood of UK extreme temperatures locally from model experiments with and without anthropogenic forcings that provide data on a relatively coarse resolution.

The HadUK-Grid data cover the entire UK and are available for the period 1960–present. Annual values of *tx01* are calculated for all the grid boxes, and a map of the trends over the observational period is illustrated in Fig. 1b. Warming trends dominate and are most prominent in the southeast, where they may locally reach 1 °C decade^−1^. Testing the significance of the trends with the Mann–Kendall test, indicates they are significantly different than zero in most regions, but not in parts of Scotland where there is a weaker warming and also areas of cooling. It should be noted that although this is a useful qualitative assessment, the trend estimates are sensitive to the start and end dates. The UK’s warmest day in a year is also estimated (Fig. 1a) after averaging daily maximum temperatures of each day over the observational area. Year 2019 has the highest UK mean *tx01* value on record, though climate models indicate that due to internal variability there is a current risk of even higher temperatures.

### Transfer functions for the estimation of local *tx01*

**Fig. 2:**
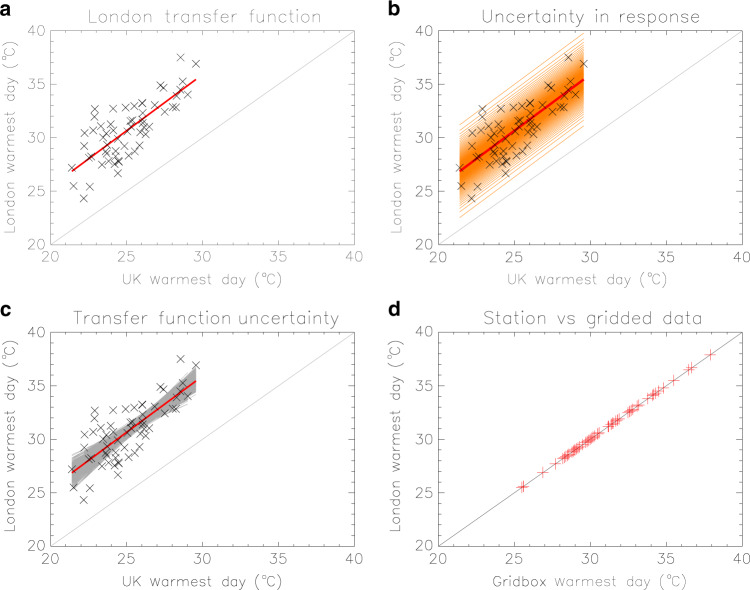
Transfer functions for the estimation of the local warmest daytime temperature (*tx01*). An example for a grid-box in London. **a** Local observations of *tx01* plotted against the UK mean observed values (crosses). A linear fit to the data (red line) represents the transfer function for the grid-box. **b** Inclusion of the confidence bounds for the response variable (orange lines) leads to a set of a 100 transfer functions in total. **c** A bootstrapping procedure applied to the observed data (crosses) provides alternative transfer functions (grey lines), used to assess the effect of sampling uncertainty. For each of the grey lines, a set of 100 transfer functions can be obtained as shown in panel **b**. **d** Observed *tx01* data from a station within the reference grid-box agree well with the HadUK-Grid data.

Even though climate models can provide reliable estimates of the UK mean *tx01*, as discussed later, their spatial resolution is still too coarse to yield local estimates on a 1-km grid. We therefore derive observationally based transfer functions to obtain local *tx01* values from the UK mean that we can later apply to model data. A simple linear fit is applied to all the grid boxes of HadUK-Grid to model the relationship between the grid-box *tx01* and its UK-mean counterpart. An example for a grid-box in London is shown in Fig. 2a. We account for the range of values of the response variable (local *tx01*) by estimating its confidence bounds for each percentile^21^ (orange lines in Fig. 2b) and so end up with a set of 100 possible transfer functions at each grid-box ('Methods'). Moreover, given the limited sample of 60 years, our analysis also investigates the uncertainty in the transfer functions, by applying a Monte Carlo bootstrap procedure that resamples the observational data. This procedure offers alternative transfer functions (grey lines in Fig. 2c), each with an associated set of 100 variants, as previously explained. Finally, it is important to establish whether grid-box temperatures on spatial scales of 1 km can adequately represent local temperatures. To this end, we compare station observations across the UK with the HadUK-Grid temperature of the grid-box where each station is located and confirm a good agreement in all cases (Fig. 2d; Supplementary Note 1 and Supplementary Fig. 1).

### The CMIP5 ensemble

Estimates of the UK mean *tx01* are next obtained from simulations with 16 climate models that participated in the World Climate Research Programme’s Coupled Model Intercomparison Project phase 5 (CMIP5)^22^. The models provide simulations of the actual climate (all forcings) under the influence of both natural and anthropogenic forcings, as well as of a hypothetical natural world without the effect of human influence. Anthropogenic forcings include historical changes in well-mixed greenhouse gases, aerosols, ozone and land-use. Natural forcings include only volcanic aerosol emissions and changes in the solar irradiance. Data from the following atmosphere–ocean coupled CMIP5 models are used in the analysis:

ACCESS1-3, bcc-csm1-1, CCSM4, CESM1-CAM5, CNRM-CM5, CSIRO-Mk3-6-0, CanESM2, GFDL-CM3, GFDL-ESM2M, HadGEM3-ES, IPSL-CM5A-LR, IPSL-CM5A-MR, MIROC-ESM, MIROC-ESM-CHEM, MRI-CGCM3, NorESM1-M.

The all-forcings experiment was also extended to the end of the twenty-first century with projections that follow the representative concentration pathway (RCP) scenarios^17^ RCP 4.5 and 8.5 scenarios. Given the substantial volume of the simulated daily data, we employ only one simulation per model and per experiment (r1i1p1), thus placing equal weight on all models. We apply a simple bias-correction to all the model data to make sure that the mean *tx01* value in the all-forcing simulations during the base period 1961–1990 agrees with the observed mean value. For consistency, we re-grid all the models on a common 60-km resolution grid, on which the observations are also available, and then mask the model data with the observations on the same grid and compute the UK-mean value. For each model, we subtract the mean observed from the mean modelled *tx01* over the base period and remove the resulting bias from the *tx01* estimates with and without anthropogenic forcings derived from the same model.

### Model evaluation

**Fig. 3:**
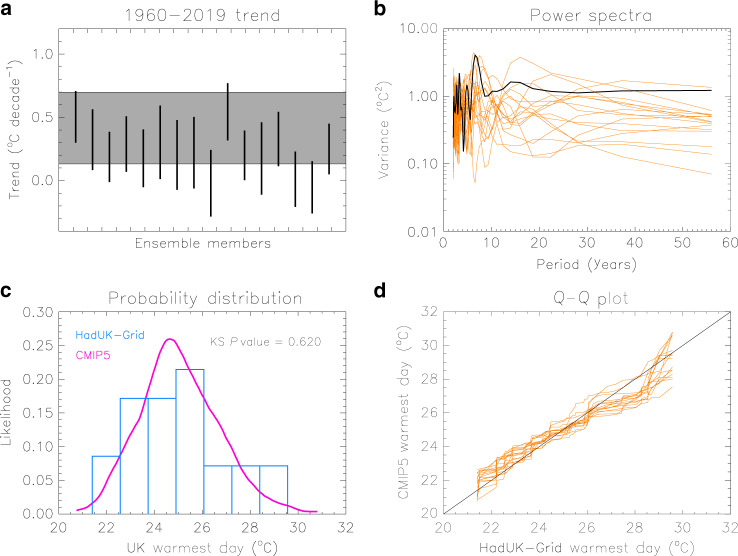
Model evaluation. **a** The ±2 standard deviation range of the 1960–2019 trend (°C decade^−1^) in the UK mean warmest daytime temperature (*tx01*) estimated with HadUK-Grid observations (grey band) and CMIP5 model simulations with all forcings (vertical bars). **b** Power spectra from detrended timeseries of the UK mean *tx01* computed with observations (black line) and model simulations (orange lines). **c** Normalised distributions of the UK mean *tx01* in period 1960–2019 from observations (blue histogram) and aggregated data from the all-forcing simulations (pink line). The *P*-value of a Kolmogorov–Smirnov test that assesses whether the two distributions are significantly different is also shown. **d** Quantile–quantile (Q–Q) plots for each of the 16 models, comparing the simulated and the observed UK mean *tx01* distributions.

Evaluation of the models against observations in attribution analyses is essential, in order to determine whether they are fit-for-purpose. Here we compare the data of the UK mean *tx01* during 1960–2019 simulated by the all-forcings experiment with observationally based data from HadUK-Grid. We apply a set of standard evaluation tests to assess how well the models represent the trends, variability and distribution of *tx01*. Results are shown in Fig. 3. First, we estimate the trends in *tx01* over the observational period and its associated ± 2 standard deviation range computed with least-square fits (Fig. 3a). The observations indicate a small positive trend, but its precise value is uncertain because of the effect of variability. Although some models produce weaker trends than the observations, the relatively short length of the observational period prevents a more detailed assessment, and since all models have a range that overlaps with the one from the observations, they are all included in the analysis. We next assess the simulated variability over different timescales with power spectra from detrended *tx01* timeseries (Fig. 3b). The observed spectrum is found to be within the range of the modelled spectra, albeit towards the higher end, though again sampling limitations need to be taken into consideration. The observed and modelled distribution of the UK mean *tx01* in the period 1960–2019 is illustrated in Fig. 3c. The modelled distribution is constructed with data from all 16 models and is found to be indistinguishable from the observed distribution, when a Kolmogorov–Smirnov (KS) test is applied (*P*-value greater than 0.1). The shape of the observed distribution (histogram) indicates that temperatures in upper tail are not well sampled because of the limited length of the record. This is also reflected in the quantile–quantile plot of Fig. 3d, which nonetheless still indicates that the models can realistically represent the distribution of *tx01*. In conclusion, the simple evaluation tests described here do not raise concerns about the ability of the multi-model ensemble to represent the UK *tx01*, but, on the contrary, indicate it is a sufficiently good dataset for the attribution analysis.

### Testing the transfer functions with the CMIP5 models

A main assumption in our methodology is that the transfer functions we derive from the observations are not sensitive to the non-stationarity of the climate to an extent that would weaken our results. Local and regional temperatures are influenced by both internal variability and external forcings, and the interplay between these two factors would be different in different periods^23^. Using the CMIP5 models, we test the effect of non-stationarities and the interplay of variability and external forcings. First, we establish that the choice of the training period for which the transfer functions are derived does not compromise the analysis. We derive a set of three transfer functions from the models, corresponding to three different scenarios:

1. Strong forcing influence: The transfer functions are derived from simulations with all forcings for future years 2020–2100, characterised by a strong anthropogenic influence.
2. Variability influence only: The transfer functions are derived from simulations with natural forcings only, i.e., in the absence of any anthropogenic influence.
3. Mixed response: The transfer functions are derived from simulations with all forcings for the period 1960–2019, i.e., the same as the observational period used in our main analysis, for which anthropogenic influence increases with time.

**Fig. 4:**
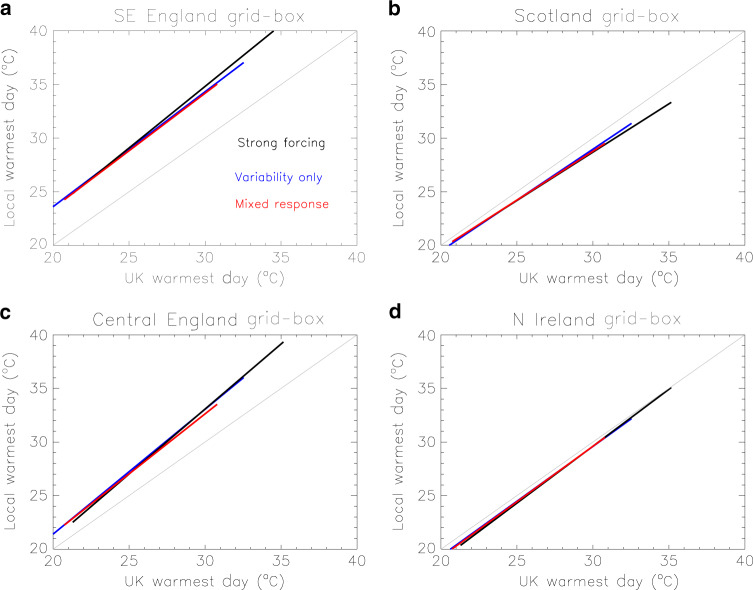
Transfer functions derived from the 16 models. Functions computed with simulations with all external forcings and training periods 2020–2100 (strong forcing) and 1960–2019 (mixed response) are shown in black and red, respectively. Functions from simulations with natural forcings only (variability only) are shown in blue. Each panel corresponds to a different grid-box.

The transfer functions obtained for four different grid boxes (in the Southeast London area, Scotland, Central England and Northern Ireland) are shown in Fig. 4. It is evident that different training periods yield similar transfer functions. The largest discrepancies are generally within a degree (in most cases much smaller) and are eclipsed by uncertainties due to sampling (Fig. 2c) and internal variability (Fig. 2b), which have been included in our approach. For example, the uncertainty range in Fig. 2c spans ~10 °C, and is accounted for by using 100 variants of the transfer function. We repeated the sensitivity tests over different grid boxes, training periods and with individual models, and found no indication of a large uncertainty due to the non-stationary climate that would adversely affect our results.

**Fig. 5:**
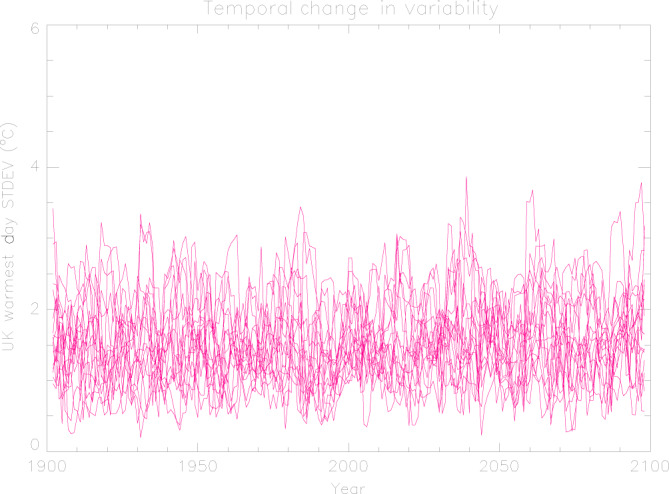
Timeseries of the standard deviation of the UK mean warmest daytime temperature (*tx01*) constructed with each of the 16 models. The standard deviation was computed in 5-year rolling windows after subtracting the forced response from model simulations with all forcings.

We also test whether internal variability may significantly change in a non-stationary climate over the course of the century. We use the multi-model ensemble mean of the UK *tx01* timeseries as an estimate of the forced response and subtract it from all the timeseries by individual models. We then compute the standard deviation in 5-year rolling windows, which provides the timeseries of the standard deviation shown in Fig. 5. The models indicate no major change in variability over the period 1900–2100. Finally, as a way of assessing the quality of the of the simulated UK mean *tx01* samples used in the analysis, we examine how different model combinations might affect the local *tx01* distributions. We find that different samples yield similar distributions (Supplementary Note 2 and Supplementary Fig. 2) and conclude that the sample choice does not introduce a considerable uncertainty.

### Attribution on local scales

We adopt a popular risk-based event attribution framework^24^, whereby *tx01* estimates from the two multi-model ensembles (with and without human influence) are used to generate probability distributions for the actual and natural climate. Local distributions are constructed on the observational (1 × 1 km) grid by applying the previously derived transfer functions to the simulated UK mean *tx01* and the uncertainty in the transfer functions is accounted for by the bootstrapping procedure. Details on the construction of the local distributions are given in the ‘Methods’.

**Fig. 6:**
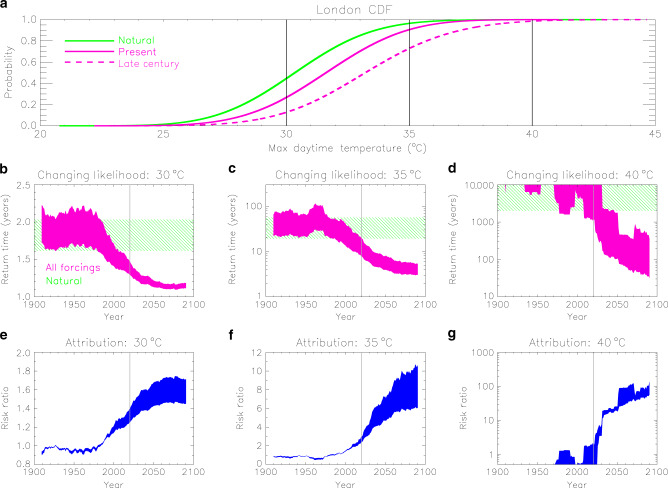
Increasing chance of high-threshold exceedance illustrated for a location in London. **a** Cumulative distribution functions of the local warmest daytime temperature (*tx01*) for the natural climate (green line), the present-day climate (pink solid line) and the climate of the late twenty-first century (pink dashed line). The 30, 35 and 40 °C thresholds are marked by the vertical black lines. Panels **b**-**d** show timeseries of the return time (inverse probability) for the exceedance of the three thresholds with all forcings (in pink). The thickness of the timeseries illustrates the uncertainty in the transfer functions used in the analysis. The expected range in the natural climate is marked in green. Panels **e**–**g** show timeseries of the risk ratio (in blue) for the three thresholds, measuring the change in the likelihood of exceeding the threshold relative to the natural climate. The thickness of the timeseries represents the 5–95% uncertainty range. The vertical grey lines in panels **b**–**g** mark year 2020 (i.e., the present climate).

Results from our analysis for a grid-box in London are illustrated in Fig. 6. For this example, the all-forcing simulations were extended to 2100 with the RCP 4.5 scenario. The cumulative distribution function (CDF) of *tx01* shifts to higher values with time, increasing the likelihood of exceeding 40 °C, which is near-zero in the natural climate (Fig. 6a). Exceeding the lower threshold of 30 °C in London is common and occurs almost every year, even without the anthropogenic effect (Fig. 6b). However, temperatures above 35 °C are now 2–3 times more likely than in the natural climate (Fig. 6f), and model projections suggest they will occur at least twice a decade at the end of the century (Fig. 6c). The likelihood of exceeding 40 °C in the reference location is still extremely low, but is rapidly increasing, with the return time falling from thousands of years in the natural world to hundreds, or even tens of years by 2100 (Fig. 6d). For London, these likelihoods could increase even further as a consequence of increased urbanisation in future or from higher rates of local anthropogenic heat release^25^, for example from wider adoption of air conditioning during heatwaves.

**Fig. 7:**
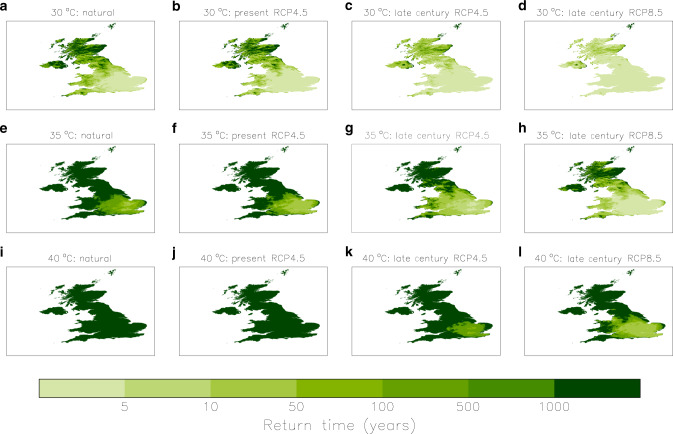
The changing likelihood of locally exceeding high thresholds of the warmest daytime temperature (*tx01*) in the UK. Maps of the return time for *tx01* going above 30 °C (panels **a**–**d**), 35 °C (**e**–**h**) and 40 °C (**i**–**l**) in the natural climate (panels **a**, **e**, **i**), the present climate (**b**, **f**, **j**), and the climate of the late twenty-first century simulated with the RCP 4.5 (**c**, **g**, **k**) and RCP 8.5 scenarios (**d**, **h**, **l**).

Repeating the analysis on all grid boxes, we also produce maps showing the return time for local exceedances of the three temperature thresholds (Fig. 7). Given the high spatial resolution of the plotted fields, certain topographic or coastal effects become evident on close inspection. Besides the noticeable contrast between warmer summers in the south and cooler in the north, the southeast England clearly stands out as the region where high temperature extremes are most likely to occur. Compared with the natural world, there are now more areas likely to see temperatures exceeding 30 or 35 °C, while the 40 °C threshold is still very rare, even in the southeast. By the end of the century, most areas in the north of the UK will also be regularly experiencing days with temperatures at least as warm as 30 °C, while crossing the 35 °C becomes common in the southeast under RCP 4.5 and over most of England under RCP 8.5. The highest threshold of 40 °C is to be exceeded at least once a century in the London area under RCP 4.5, and several times a century over most of southeast England under RCP 8.5. The effect of the uncertainty in the transfer functions has also been assessed (Supplementary Note 3 and Supplementary Figs. 3 and 4), and although it may change to some extent the intensity and spread of the map features in Fig. 7, the main conclusions still hold.

### Chances of exceeding extreme thresholds anywhere in the UK

**Fig. 8:**
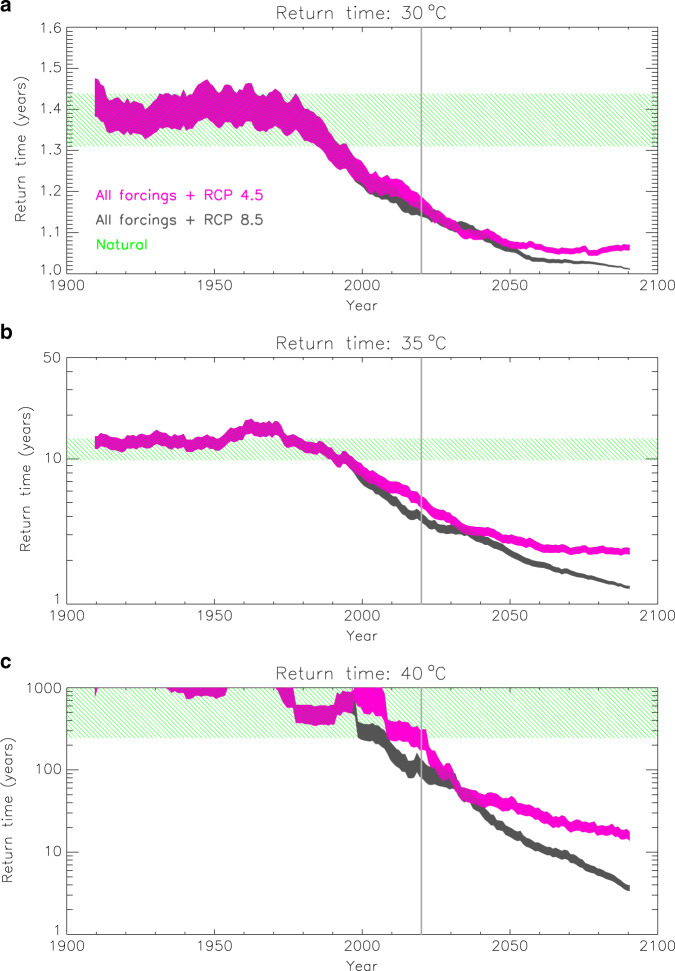
The increasing likelihood of exceeding high temperature thresholds anywhere in the UK. Timeseries of the return time for observing temperatures in the UK above **a** 30 °C, **b** 35 °C and **c** 40 °C with all forcings and future projections following the RCP 4.5 (in pink) and RCP 8.5 (in grey) scenarios. The thickness of the timeseries illustrates the uncertainty in the transfer functions used in the analysis. The expected range in the natural climate is marked in green. The vertical grey lines mark year 2020 (i.e., the present climate).

We finally compute the likelihood of exceeding an extreme threshold in a given year, not at a specific location, but anywhere in the UK. The chance of rising above, for example, 40 °C, the most extreme threshold examined here, might still be very low for a given location, but has been increasing in most areas under the influence of warming trends (Fig. 1b). When all grid boxes are examined together, the likelihood of getting at least one grid-box that exceeds 40 °C in a specific year is expected to be higher than a local likelihood. The CMIP5 models provide a large number of alternative representations for every year. Each representation may yield a hit, i.e., at least one location where the reference *tx01* threshold is exceeded, or not, and the likelihood of exceeding the threshold anywhere in the UK may thus be determined by the count of hits ('Methods'). Figure 8 depicts timeseries of the return time for different threshold exceedances and its expected range in the natural climate. Rising above 35 °C is estimated to occur once every 5 years at present and almost every year by the end of the century (Fig. 8b). Also, the probability of recording 40 °C, or above, in the UK is now rapidly accelerating and begins to rise clearly above the range of the natural climate (Fig. 8c). The return time for the 40 °C threshold is reduced from 100–1000s of years in the natural climate to 100–300 years in the present climate and to only about 15 years by 2100 under the medium-emissions scenario (RCP 4.5) and 3.5 years under the high-emissions scenario (RCP 8.5).

## Discussion

Our study demonstrates that human-caused climate change has set hot-day extremes in the UK on a course towards temperatures that would be too high to be observed in the natural climate. As the warming continues, new records are expected in coming decades, with the most severe extremes likely to occur in the southeast of the UK. Our attribution analysis derives local information from observations rather than regional models and investigates high-impact extremes that could break out anywhere in the UK rather than in prescribed locations. There are, of course, uncertainties in our analysis, some of which we have explored and tried to address, including uncertainties in the transfer functions and the limited number of years they are based on, or the limited number of models employed and their ability to represent the UK climate. Although the transfer functions make a distinction between urban and rural locations, large future changes in the UK’s urban landscape could present a caveat in the analysis, though this is likely to affect only a small fraction of grid boxes. Future probability estimates are found to be sensitive to the choice of the RCP in the model simulations. Here, we estimate the future likelihood of extremes under both a mid- and high range RCP scenario. However, if emissions are reduced in line with the Paris climate agreement, the future probabilities are expected to be lower. Despite these uncertainties, our analysis still clearly establishes the nature of already realised and future changes in extreme temperatures including their spatial characteristics, information that can help the UK plan its resilience to heat extremes.

## Methods

### Transfer functions

The local and UK mean *tx01* is computed from the observations for every year in the period 1960–2019. For a given grid-box, we represent the dependence of the local *tx01* on the UK mean with a simple linear model:

$$tx01\left( {{\mathrm{local}}} \right) = \alpha _0 + \alpha _1tx01\left( {{\mathrm{UK}}} \right).$$

The linear regression is fitted to the *n* = 60 observed annual values of *tx01*(local) and *tx01*(UK) and ordinary least squares are used to estimate the coefficients *α*_0_ and *α*_1_.

If **y**_*i*_^obs^ and **y**_*i*_^fit^ denote the observed and fitted values of *tx01*(local) in year *i*, and SSE the sum of squared errors:

$${\it{{\mathrm{SSE}}}} = \mathop {\sum}\limits_{i = 1}^n {\left( {y_i^{{\mathrm{obs}}} - y_i^{{\mathrm{fit}}}} \right)^2} ,$$

then the confidence interval for the response variable and the (1 + *p*)/2 quantile of the *t*(*n* − 2) distribution is estimated^19^ as:

$${\mathrm{ \pm }}t_{{\mathrm{(1 + }}p{\mathrm{)/2}}}\sqrt {\frac{{{\mathrm{SSE}}}}{{n{\mathrm{ - 2}}}}} \sqrt {{\mathrm{1 + }}\frac{1}{n} + \frac{{\left( {x_i^{{\mathrm{obs}}} - \overline X } \right)^2}}{{{\it{{\mathrm{SXX}}}}}}} ,$$

where **x**_*i*_^obs^ denotes the observed value of *tx01*(UK) in year *i*, $\overline {\mathrm{X}}$ the mean of the observed *tx01*(UK) values and SXX is calculated as:

$${\mathrm{SXX}} = \mathop {\sum }\limits_{i = 1}^n \left( {x_i^{obs} - \overline X } \right)^2.$$

The confidence bounds are represented by very shallow hyperbolas that can be almost perfectly approximated by straight lines, as done in this study. Using the best fit to the observed data and the 1st to 99th percentiles for the estimation of the uncertainty range for the response variable, we end up with a set of 100 transfer functions per grid-box. Therefore, when we apply the transfer functions to a model simulated value of *tx01*(UK), we obtain 100 values that represent the possible range of the *tx01* at the reference location.

### Uncertainty in the transfer functions

Although the 60-year long observational dataset used to derive the transfer functions is deemed large enough to provide reliable estimates of the linear fits at every grid-box, sampling uncertainty will still have some effect on the analysis results. This kind of uncertainty is commonly accounted for by a simple a Monte Carlo bootstrap procedure^26^ that we also employ here. The procedure involves random resampling of the 60 annual pairs of *tx01*(UK) and *tx01*(local) and deriving a new set of transfer functions from the resampled data. Multiple resampling provides multiple sets of transfer functions. Each set, as explained next, can provide an estimate of the likelihood of the local *tx01* exceeding a certain temperature threshold and by repeating the calculations with all the bootstrapped sets of transfer functions, we obtain multiple estimates of the likelihood, which enables us to estimate its uncertainty range.

### Estimation of the local *tx01* probabilities

For every year of each experiment we obtain samples of 1600 *tx01* values for every grid-box (16 CMIP5 models that provide estimates of the UK mean *tx01* × 100 transfer functions). We further increase the sample size in the all-forcings experiment to 32,000 by calculating the probabilities in 20-year rolling windows during the period 1900–2100 (i.e., in time segments 1900–1919, 1901–1920, …, 2081–2100). We select years 2011–2030 to represent the present-day climate and 2081–2100 to represent the late twenty-first century climate. Future probabilities are estimated with both RCP 4.5 and 8.5, whereas only simulations with RCP 4.5 were used to estimate present-day probabilities. For the natural world, we aggregate all simulated years (1900–2005), assuming that the natural climate is stationary in the long run, which yields samples of 169,600 *tx01* values (16 models × 100 transfer functions × 106 years) for every grid-box. The resulting samples provide estimates of the likelihood of exceeding the pre-selected thresholds of 30, 35 and 40 °C. Given the large sample sizes, probabilities are computed by a simple count of threshold exceedances. Using alternative sets of the transfer functions from the bootstrapping procedure described earlier, we re-calculate the probabilities multiple times and estimate their 5–95% range to account for the uncertainty in the empirical relationships.

### Probability of exceeding a threshold anywhere in the UK

The 16 CMIP5 models provide 320 simulated years in consecutive 20-year rolling windows. The sample increases to 32,000 when we apply the set of 100 transfer functions for each grid-box to obtain high-resolution annual maps of the *tx01*. Counting how many of these 32,000 maps include at least one location where the reference threshold is exceeded, allows us to the calculate the probability estimate. As before, the probability is recomputed with alternative sets of the transfer functions to assess their uncertainty. The natural probabilities are also aggregated to provide the 5–95% range for the natural climate.

## Supplementary information

Supplementary Information

## Data Availability

The HadUK-Grid temperature data and station temperature data from the Met Office Integrated Data Archive System (MIDAS) that support the findings of this study are available from the CEDA Archive, http://archive.ceda.ac.uk. The CMIP5 simulated temperature data that support the findings of this study are available from the Earth System Grid Federation (ESGF) Archive, https://esgf.llnl.gov/.

## Code availability

IDL code used for the analysis is available upon request.
