# Supplementary material for: The increasing likelihood of temperatures above 30 to 40 °C in the United Kingdom
Source: Nat Commun. 2020 Jun 30;11:3093. doi: 10.1038/s41467-020-16834-0 (PMC7326920; doi:10.1038/s41467-020-16834-0)
Supplement: Supplementary file 1 — Supplementary Information [file 41467_2020_16834_MOESM1_ESM.pdf]

Supplementary Information for:

The increasing likelihood of temperatures above 30 to 40 ^{0}C in the United Kingdom

Christidis et al., Nature Communications

Supplementary Note 1. Representation of local temperatures on the 1×1 km grid.

The high spatial resolution employed in our analysis should be able to represent local temperatures well, given their large spatial correlation scales, but we still need to test this assumption to make sure that variations within a grid-box do not introduce biases. We therefore compare *tx01* values during 1960-2017 from 16 observation stations across the UK with the HadUK-Grid values of the station grid-box. The station data are retrieved from the Met Office Integrated Data Archive System (MIDAS) available from http://archive.ceda.ac.uk/. The highest UK temperature of 38.7 ^{0}C recorded at the Cambridge Botanic Garden station on 25 July 2019 is identical to the temperature of the corresponding grid-box of HadUK-Grid. Supplementary Figure 1 reveals a good agreement between station and grid-box *tx01* values for all the stations examined.

Supplementary Note 2. Model sample uncertainty.

It is essential that the samples of the simulated UK mean *tx01* values used in the analysis are of good quality. These samples come from simulations with the 16 state-of-the-art CMIP5 models that provide a sufficiently good representation of the UK mean trend in *tx01*, its variability and overall distribution. We tested whether the attribution results might be sensitive to the model ensemble used in the analysis and examined how different model combinations might affect the local *tx01* distributions. Supplementary Figure 2 illustrates different versions of the CDF for a grid-box in Heathrow constructed with all possible 15-model combinations of the CMIP5 models. Tests in other locations provide similar results. We find that the different samples yield similar CDFs, indicating uncertainties in the return time of up to a few years. These are much smaller than the uncertainties from the transfer functions that have a range of tens, or even hundreds of years (Fig. 6 b-d).

Supplementary Note 3. Uncertainty in return time maps.

The return time maps for *tx01* values above pre-specified thresholds shown in Fig. 7 are produced with the original set of the 100 transfer functions derived from HadUK-Grid. To test the effect of the uncertainty in the transfer functions, local return times were re-estimated multiple times with alternative sets of the empirical relationships obtained with bootstrapping (details in Methods) and the 5-95% uncertainty range was estimated on every grid-box. As the bootstrapping procedure is computationally expensive, late century probabilities are estimated here only with the RCP 4.5 scenario. The maps corresponding to the 5th and 95th percentiles are shown in Supplementary Figures 3 and 4 respectively. The main features of the maps in Fig. 7 are still present, though their extent varies, as expected. Changes in small-probability events (like the exceedance of 40 ^{0}C) are more pronounced, as the estimation of their return time entails larger uncertainties. Nevertheless, the overall good similarity between the maps across their uncertainty range makes them particularly useful as an aid to decision-making.

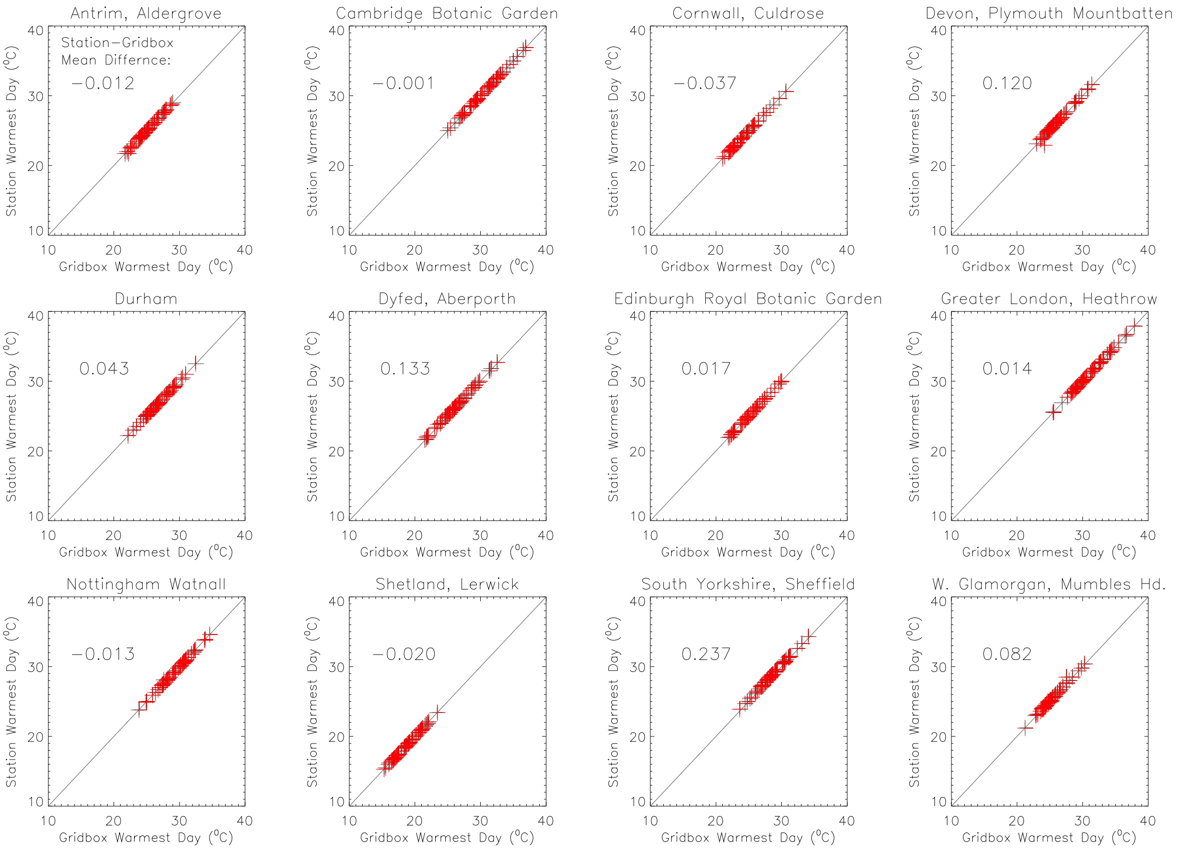

Supplementary Figure 1. Station vs grid-box data. Observed station data of the warmest daytime temperature (*tx01*) for years 1960-2017 plotted against HadUK-Grid data at the grid-box where the station is located. Each panel corresponds to a different location. The mean difference between the station and grid-box estimates of *tx01* is also shown.

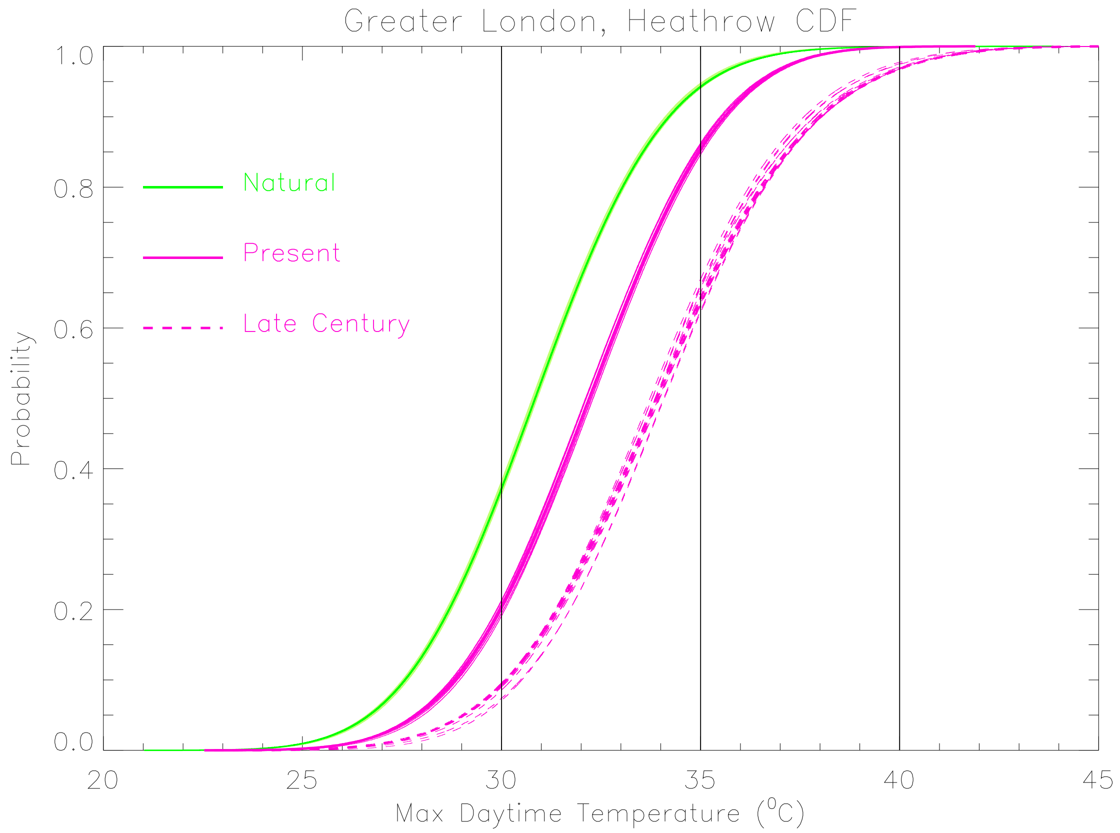

Supplementary Figure 2. Cumulative distribution functions (CDFs) of the warmest daytime temperature (*tx01*) at Heathrow for the natural climate (green lines), the present-day climate (red solid lines) and the climate of the late 21st century (red dashed lines). Multiple CFDs were derived for each case from all possible 15-model combinations. The 30, 35, and 40 ^{0}C thresholds are marked by the vertical black lines.

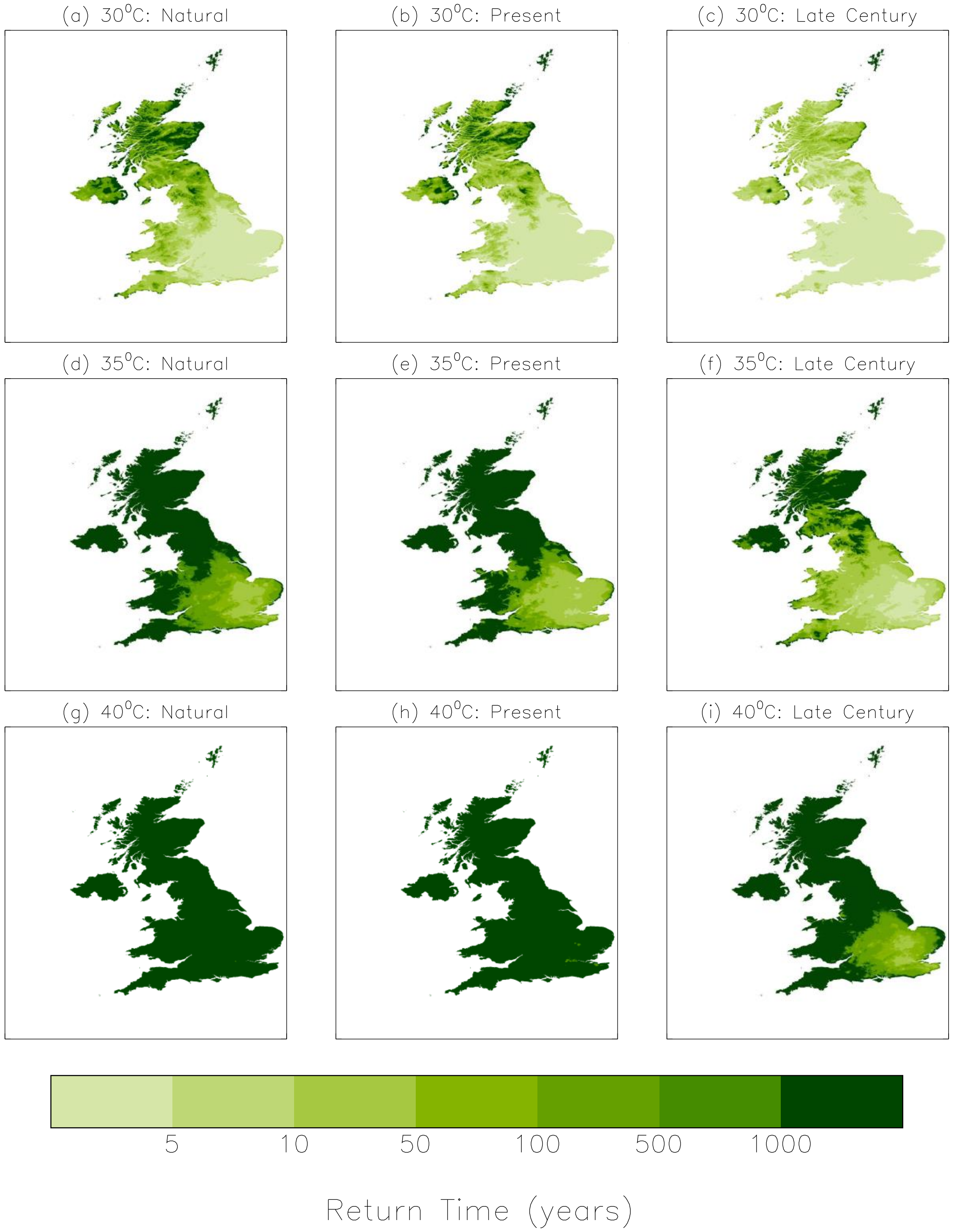

Supplementary Figure 3. The effect of uncertainty in the transfer functions on local estimates of the return time for the warmest daytime temperature (*tx01*) going above high temperature thresholds. Maps similar to the ones shown in Fig. 7 are plotted here, but for the 5th percentile of the return time, while future probabilities are only estimated with the RCP 4.5 scenario.

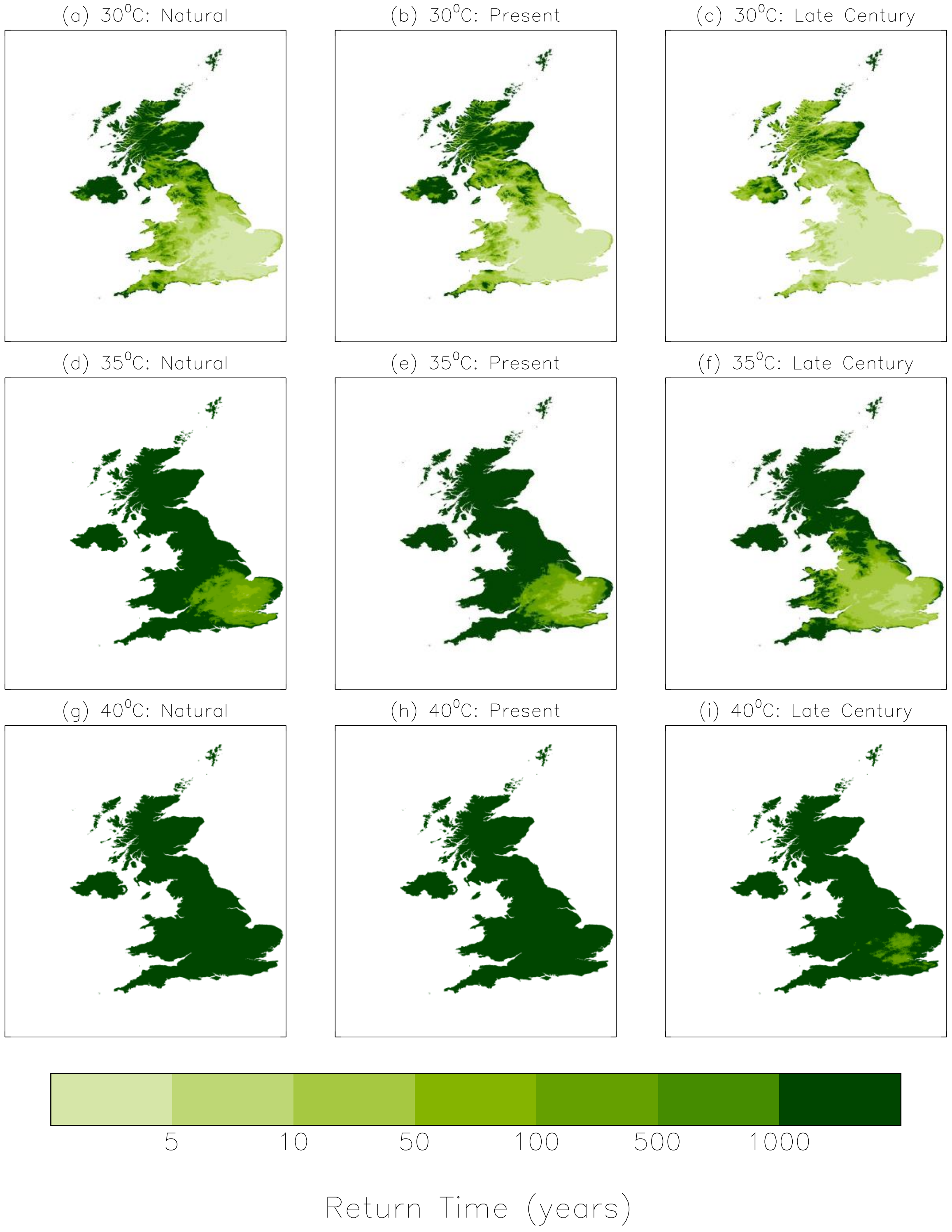

Supplementary Figure 4. The effect of uncertainty in the transfer functions on local estimates of the return time for the warmest daytime temperature (*tx01*) going above high temperature thresholds. Maps similar to the ones shown in Fig. 7 are plotted here, but for the 95th percentile of the return time, while future probabilities are only estimated with the RCP 4.5 scenario.
